# Intelligence

**Published:** 1828-03

**Authors:** 


### INTELLIGENCE.

### LIBEL AGAINST THE EDITOR OF THE LONDON MEDICAL AND PHYSICAL JOURNAL.

This cause, which has been so long postponed, at length came on in the Court of King's Bench, on Monday, the 18th of February. We shall subjoin an account of the proceedings, taken by a short-hand writer employed for the purpose, and afterwards make a very few remarks.

### Macleod v. Wakley,

*(Tried before* Lord Tenterden *and a Special Jury.)*

Mr. Pattison opened the pleadings.

Sir James Scarlett then proceeded to state the case on the part of the plaintiff as follows:—

May it please your Lordship; Gentlemen of the Jury; The plaintiff in this case, Dr. Macleod, is a physician, who for the last ten or twelve years has been practising in this metropolis: he is a gentleman exceedingly well known both by the circle in which he moves as a medical man, and by the public who take an interest in medical subjects, by his having become the editor of a publication that has long had great reputation, called the London Medical and Physical Journal. He was the joint editor with Mr. Bacot up to a certain period, and from that period he became the sole editor. The object of that work is to take notice of such publications of new medical facts as may be interesting to the profession and useful to the public, and also occasionally to make some comments upon new medical information that is derived from public sources, for the purpose of instruction, and informing the profession of what is passing; in short, to record, as it were, the living history of medicine.

Gentlemen, I should inform you, in order to explain the matter of the libel which Dr. Macleod calls on you this day to consider, that he had published, in the month of December 1825, a case of the cure—real or supposed —of a disorder which is called aneurism. [Here the learned Counsel described what aneurism meant, and alluded particularly to Mr. Wardrop's proposal to tie the artery beyond the tumor. He then proceeded:] This certainly has been attempted in one or two instances before, but was supposed to have failed; but Mr. Wardrop having published this case as one of success, it was noticed by Dr. Macleod in terms of very considerable approbation: he begins by stating "This is one of the boldest operations to be found in the records of modern surgery," and the case is "unquestionably very interesting and important." The whole case is then given sufficiently to make anybody understand it, and to draw the public attention to it, occupying a space, I think, of no less than one—two—three pages, nearly three pages and a half of this Journal. That was in the year 1825. In the latter end of 1826, another case occurred, which Mr. Wardrop had published in a work called the Lancet, which is published by the defendant, (I believe he is the proprietor of it also,) of very great circulation: it is published in a very cheap form, being sixpence a number, and it combines with a notice of medical facts a species of humor and satire which, as you all know, render literary performances the more acceptable.

[Here some interruption took place in the Court.]

Gentlemen, I was stating to you a proposition, in which I hope you will agree, that a performance—whether it be of a scientific nature, or of a purely literary nature,—that is seasoned with a portion of satire and of personal allusion, generally speaking, (for what cause I do not pretend to know,—some cause founded in a principle of human nature, I suppose,) has more circulation than any other; and the case of aneurism that had occurred in the following year having been published by Mr. Wakley in the Lancet, had given it sufficient circulation, and there was no occasion for Dr. Macleod, therefore, to notice it. However, in a number of his work published in April 1827, it was noticed by a writer who had made him an original communication, a gentleman (Mr. Travers) who had stated several cases of disorders of arteries, of which this is one, and alluded to this case of Mr. Wardrop; so that it was noticed in that number. But it so happened, before the number went to the press, that the last patient on whom the experiment had been tried by Mr. Wardrop died, and, upon her death, an examination had been made into the particular part where the aneurism had taken place, in order to ascertain the success of the operation; because, although a patient recovering from an operation is undoubtedly, as long as the patient lives, the best evidence you can have of the success of it, yet, as persons do not know the secrets of nature, the most learned anatomist has still something to learn on that subject: it sometimes happens that nature does the work which we ascribe to the artist; and therefore when the lady—I think it was a female—died, on whom the second operation had been performed by Mr. Wardrop, an opportunity being given in the hospital to make a dissection of the particular part, it was thought very important by the surgeons to ascertain whether or not the ligature which he had applied had really been a ligature of the artery, or of some other membrane.

Now, I should inform you that it is a known fact among medical men, that where the artery is completely tied, so as to prevent all communication of the blood from one side to the other by the ligature, that the effect of it is (and that is the object of the cure in aneurism,) to cut it off as it were,—the effect is to obliterate altogether the artery, so that the two extremities become sacs, as it were, at each end, and there is no communication of the blood from one to the other: if any connexion remains between them, and that connexion is pervious so that an instrument can pass through it, that is a proof that the ligature has not been complete, and has not been put there. On the dissection of this part of the frame of the patient, who was supposed to have recovered of the aneurism, but was still a patient in the hospital, it was completely ascertained that, in point of fact, the artery, where the supposed ligature had taken place, was in a perfect state—was pervious, and therefore the blood had flowed through it from the heart to the head just as before: consequently, that the cure must have taken place from some other cause,—either by nature or from some other cause, but not from this attempt. Now this was a fact so important, being an original fact, that Dr. Macleod thought it right—that it was his duty, to communicate this to the public, and he does so in this number, and in this form. You see the article from Mr. Travers had appeared in the body of the work; before it went to the press, this fact being ascertained, and Mr. Travers having spoken in commendation of the supposed new practice, relying on this case and the former case, this notice is taken by Dr. Macleod of the event that I have just now described to you:—"Dissection of one of the cases of aneurism in which the carotid artery was supposed to have been tied beyond the tumor.—Our readers are probably aware that it was proposed by Dessault to tie the artery, in certain cases of aneurism, beyond the tumor, and that this operation was actually performed by Deschamps and Sir Astley Cooper, but, proving unsuccessful with them, never became generally adopted. Allusion is made in the present number of the Journal to Mr. Wardrop's attempt to revive this method of operating, and we therefore think it right to make our readers acquainted with the state of parts as discovered on the post-mortem examination of one of the recent cases. The patient alluded to died last week, and the body was examined on the 23d, when it was found that the carotid artery was pervious and undisturbed, presenting one continuous tube throughout, there being no unusual appearance and no aneurism. The heart was affected with hypertrophy;" of which the patient, I fancy, died. "Mr. Travers, with reference to the alleged success of this method, remarks (page 331,) that it will be of much importance, 'if borne out by similar results,' and we have given the above details because it is obviously of great importance that surgeons should be able to form a true estimate of the value of any proposed method of treatment as soon as possible, that it may either be rejected or adopted according to circumstances. We are quite aware that mistakes will sometimes happen even in the hands of skilful surgeons, and it is this consideration which has induced us to withhold numerous other instances of unfortunate operations, which have been transmitted to us for the purpose of publication, because they have not, like the present case, been connected with any important practical question."

Now, there is no doubt that the question was of very great practical importance to all surgeons, seeing there was one case that was supposed to be successful, and another case that had also been supposed to be successful, but on dissection had turned out not to be the case supposed,—it was very important that they should inquire into the demonstration of the fact presented, in order to ascertain what sort of reliance might be placed on the recommended operation; because there is no doubt the operation is very dangerous in its nature: the experiment is hazardous, and, unless the cases were multiplied on each other, and should present a tolerable ground of success, it would be hazardous to attempt it in any particular case.

Gentlemen, this is the whole that Dr. Macleod has done, and you will judge whether he has deserved the attack made on him by the author of the Lancet. Dr. Macleod publishes his Journal in this form, with a yellow leaf, you see, by which the author of the Lancet thinks fit afterwards to distinguish him. In the month of May, 1827, the very following month after this April publication, comes this passage on Dr. Macleod: "We must again return"—Now, you see he may be said to be in some degree—although I do not believe that he does so consider himself,—in some degree as a rival editor: rivals may at least treat each other with civility; there is no occasion to be personally rude when they enter into an honourable course of emulation; they may both serve the public. What is the character of the Lancet I do not pretend to know, but the title he assumes, I believe, is understood generally to signify that he is a great *scarifier;* and therefore the Lancet is the title of the work adopted by him for the express purpose of showing that he means to use the lancet rather freely in his commentaries, as well as his surgical operations.—"We must again return to the April number, for the purpose of noticing two papers which refer to the recent operations for the cure of carotid aneurism by tying the vessels beyond the tumor." He then takes notice of the first case of Mr. Wardrop, which was published in November 1825, as I have told you, by Dr. Macleod. He says this, "the first case successfully treated after this method was published by Mr. Wardrop in Part 1st, Volume 13, of the Medico-Chirurgical Transactions, on the 5th of July, 1825, and was copied into the Lancet of December the 31st, 1825. The second case treated by Mr. Wardrop appeared originally in the Lancet, December the 23d, 1826. The third case, treated by Mr. Lambert, was published in this Journal of March the 24th, 1827; during the whole of which period not one word has been said of these most important operations in the yellow Journal, by that exceedingly sagacious, active, and intelligent editor, Roderick Macleod."

We shall see, by and by, whether he means to use these words, which are complimentary, in an ironical sense or not. "But, on the 23d of March, the subject of Mr. Wardrop's second operation having died from an enormous hypertrophy of the heart, Roderick, in his April number, presents his readers with his first notice of either of these operations in the following account of what he calls the 'dissection' of the body. This account we will here insert, italics, capitals, and all; in fact, without altering either word or letter."

Now here he begins to inform his readers *of what he must have known was a direct and positive falsehood.* You see his object is to show that Dr. Macleod, being the editor of a Medical Journal, conducts himself with partiality and injustice, for that he omits to notice a successful case of an operation; but that, when he has discovered an unsuccessful case, he then maliciously publishes it. I have read to you the publication of Dr. Macleod, in the number of the year 1825, *which was actually antecedent to the notice taken of it by the Lancet, according to his own account* If this gentleman reviews the works of other people, he is bound to read them—I mean in point of morality. I believe he may justify himself by many other reviewers, who review works they have not read. I do remember, not very long ago, a very celebrated reviewer having reviewed a work of Mr. Buckingham's—his Travels into Asia Minor, and it was quite clear the reviewer had so misrepresented certain material facts stated, and which he said he had borrowed from the book, that he either had not read the work, or, having read it, he had falsified the statement in his review. One of two things must be charged on the author of the Lancet,—either he is deceiving his readers, by informing them he reads others' works on which he makes comments, or, having read these works, he states a deliberate falsehood, for the purpose of calumniating the author! Then he goes on, —"Now we really think that Roderick will be most heartily despised when we come to explain the matter a little, and when we reflect on what is his duty as a medical journalist." You know the old saying: we all of us can see our neighbour's faults, but our own are in a wallet behind our backs. This medical journalist is going to tax another for a fault of which he himself happens to be guilty. "Mr. Wardrop's first operation was read to the Medico-Chirurgical Society, July the 25th, 1825; his second operation published in December 1826; and Mr. Lambert's in the Lancet of March the 4th, 1827; yet, during the whole of this protracted period, Roderick keeps his readers in perfect ignorance of these most important and invaluable additions to our stock of surgical information; but no sooner does one of these patients die of another disease than this precious Macleod (or gentlemanly Macleod, as we one day heard a Scotch blockhead call him,) brings out the above history of the dissection, from which it is impossible to understand whether he wishes to convey to his readers an impression that it refers to the first, second, or third case."

Now that is a gross misstatement, because he speaks of it as one of the *recent* cases mentioned by Mr. Travers, who in the body of the work had alluded to that case.—"His reason for publishing this paper on the 1st of April, 1827, it appears is 'because it is obviously of great importance that surgeons should be able to form a true estimate of the value of any proposed method of treatment as soon as possible.' Indeed, Roderick! if you consider this a sound maxim, why did you not allude to Mr. Wardrop's successful case of 1825, until April 1827, in which time you had ejected some two dozen or more of your yellow funguses, without marking upon either of them the slightest notice of this most important improvement in surgery, which, according to yourself, should be known 'as soon as possible.' *There is a something in this conduct far too detestable to be adequately described in print.*" You will have a description of it by and by in another way.—"Such conduct as one would scarcely expect from a 'gentlemanly editor,' who is anxious that his readers should be made acquainted with pathological facts 'as soon as possible;' but it is what we should anticipate from an editor who places all his reliance for support on the corrupt patronage of hospital surgeons, and who *appears to hate, mortally hate, not only general practitioners, but all those 'factious and disaffected persons' who have espoused their cause;* and it is well known that Mr. Wardrop is one of those persons."

I beg your attention now, gentlemen, for a moment to this: what a serious charge it is to make on a professional man, that he hates general practitioners. The author of the Lancet knows as well as any man to whom he addresses this charge. A medical man, like men in every other profession, depends on his reputation, and very often for his success in life on the kind feelings and the good opinions of the members of his own profession; and to publish of him that he hates them generally, or hates any class of them, is undoubtedly published for the purpose of exciting a hostile feeling in the body at large towards the individual, and which is one of the most injurious aspersions that could be made; and Dr. Macleod, among the bitter remarks that have been made on him here, and the ridicule thrown on him, feels more deeply than any thing else that attack: one so utterly unfounded and untrue, that I cannot but think you will agree with me that every man of feeling and honourable mind must be highly sensible and feel indignant at such an imputation being cast on him.

[The Counsel then proceeded to read the other parts of the libel.]

Now, gentlemen, then, what is the charge?—to say nothing of his calling Dr. Macleod a blockhead—to say nothing of the contempt with which he treats him, speaking of his practice as detestable—speaking of his hatred to the members of his own profession,—looking at that individual charge he makes against him, he says, you, who are a medical journalist, who say it is your duty to make the earliest communication to your profession, have wilfully concealed an important medical surgical discovery, made by Mr. Wardrop, and published by him in 1825,—you have published a dozen numbers since, or two dozen—you pass it over in silence, and then, when one of his recent instances, in your opinion, turned out to be unsuccessful, and the patient died, although she died of another disorder, you have maliciously published that, in order to point out the unsuccessful operation of this gentleman, against whom you have some malice or enmity, after having allowed the other successful cases to sleep for two years.

I cannot conceive a charge more serious than this; and it is made against Dr. Macleod by the author of the Lancet, who has thought fit to publish it *in daring violation both of truth and honesty.*

Then he goes on to state—"His plan, therefore, towards those whom he dislikes seems to be to publish no account of their success, and to fabricate cases of failure as soon as possible." So that he also charges him here with a fabrication—that this case of failure is a fabrication. Now you come to understand it, he does not even state it to be a failure: what he states is this, the patient died of another disorder—both parties agree in that;—all Dr. Macleod published was this, when she died it was thought useful to examine the supposed site of the aneurism, and it was discovered there really had been none: it was another disorder mistaken for it. The artery supposed to have been tied by Mr. Wardrop, had never been tied at all; and therefore some other membrane must have been tied by him, which he had mistaken for the artery, because the artery was found in full play, pervious, and capable of conducting the blood from the heart to the head, as it had done before. He therefore fabricates no case.

Now, Dr. Macleod complains before you, a jury of his country, that, for the purpose of conveying an imputation on his honour,—upon his character as a medical man, and on his science, and on his impartiality and justice as an editor, that the *author of the Lancet has himself fabricated falsehoods—notorious falsehoods, and published them to the world as the foundation of the charge!* What does the author of the Lancet say? He says by his plea "not guilty!" He does not justify—he does not state that he had even any colour for the publication. He leaves you, then, to judge,—he leaves you to judge of his motive in making the publication. Now, what motive can a man have, gentlemen, for publishing that which is notoriously false in order to calumniate another man? Will he say that he was not in the habits of reading this Journal;—why, he makes it a subject of criticism! What right had he to say, if he was not in the habits of reading it, that a publication of Mr. Wardrop's had not taken place in it? Is a man to say to the public, I know such an author has not published in his Journal such a work, and then to say afterwards, "Upon my word, I have not read the Journal, therefore I was mistaken!" Is that an apology? It is an apology *a man of honour would not make*, and a court of justice would not receive. He holds it out here he has read it. To the reader who has not read this work (Dr. Macleod's,) and who has read his work with a conviction that he has discharged his duty as editor, the belief must be that Dr. Macleod has never published this case at all, because he not only wished to suppress the facts which conducted another man to fame, but had fabricated a case of failure, and published a falsehood in order to do him an injury afterwards.

Now, gentlemen, Mr. Wakley, by his plea, does not pretend to say any of the facts he states are true; and, consequently, if you think them libellous,—(and I think, gentlemen, I should insult your understandings by supposing you could doubt for a moment they are,) the only question is, what estimate you will give, by your damages, of the nature of this libel. Or he will say, perhaps, it was not malicious,—very likely my friend, Mr. Brougham, will say

"It was certainly wrong, I cannot justify it. You cannot suppose he had any malice in making the publication,—he had nothing but the public good in view;—he did not intend to laugh at Dr. Macleod,—he did not intend to ridicule him,—he did not intend to bring him into contempt,—he did not mean to charge him with falsehood,—but at all events he was not malicious."

Now, gentlemen, I will take that argument (if it is my friend's argument) out of his mouth; because I will show what his real intention is. What think you of a publication I am going to read you of this defendant? I told you he used the Lancet as a scarifier; it is not the only scarifier he possesses;—my friend, Mr. Brougham, shall see how HE is held up!"

[Here Sir J. SCARLETT was interrupted by Mr. BROUGHAM, who wished to prevent him from reading a notice in the Lancet of the preceding Saturday, stating that it was a "most injudicious remark on the trial, but not evidence." This objection was over-ruled, and Sir J. Scarlett proceeded—]

There is a numerous audience collected in this Court, for the purpose of hearing Mr. Brougham; and the readers of the Lancet, who, I dare say, now surround me in this Court;—they, as well as Mr. Brougham, are aware of what I am going to read to you. I am going to read it for the purpose of showing that the object of this publication *was direct, undisguised malice, and nothing but malice, against Dr. Macleod!—the object of the defence is malice!*— His own lancet is his scarifier; but he has also another! "To the Readers of the Lancet. Macleod *v.* Wakley. The 'yellow Goth' will be scarified by Mr. Brougham on Monday next, the 18th instant, in the Court of King's Bench, Westminster." (*Loud laughter.*) This is the mode in which a physician, a man of honour, a gentleman, is treated in a publication which has no right, that I know of, to meddle with his name except as a literary man, and which ought to treat the subjects which it notices at least with truth, if not with candour. Having just cause to complain of the conduct of this Journal, when the gentleman brings an action in order to dare him to the proof of what is there stated for the purpose of calumniating his character, (the only remedy a man has—the only lawful remedy,) he appears before a jury of his country, and calls on the defendant to say, "Is it true or false? I dare you to the proof." A man who pleads the "general issue" is generally supposed to intend to make an apology,—to say "I have done this through haste—I have done it through intemperance—it was in an unguarded moment—I express my contrition—I am sorry for it." And he generally gives instructions to his Counsel to say, "As I cannot make a defence on the merits, and the verdict must be against me, I must beg of you to make the best case you can to the jury to mitigate the damages." Not so the author of the Lancet: he begins *by falsehood and by malice*, and ends by putting on my friend, Mr. Brougham, (who can do any thing—either palliate or *scarify*, just as he pleases,) the odious office of being his scarifier today, and invites all the readers of the Lancet to fill this Court with an audience as numerous as it could hold for the purpose of hearing Dr. Macleod scarified!—cut to pieces!—anatomised!—and dissected!—for the purpose of hearing every thing that an ingenious mind can suggest from any one of the publications I shall read to you, or torture into something like a sarcasm, that when this gentleman goes from the Court, whatever damages you may give him, at least the author of the Lancet may have the consolation of knowing he still goes with an injured sensibility and a wounded mind. That is the feeling of the author of the Lancet. You will hear how my friend, Mr. Brougham, will acquit himself of his scarification.

[Evidence was then called to prove matters of form.]

The notice of Mr. Wardrop's first operation was then read from the London Medical and Physical Journal.

Mr. BELLAMY:—"The number for December, 1825, page 496,—it is No. 15,—"A Case of Carotid Aneurism successfully treated by tying the Artery above the Aneurismal Tumor, by Mr. Wardrop. This is one of the boldest operations to be found in the record of modern surgery, and the case is unquestionably very interesting and important. It is remarked by the author, that in those cases where it is impossible to reach the artery between the heart and the aneurismal tumor, (instances of which are by no means very rare,) it has been customary to leave the patient to the horrors of his fate. But, says Mr. Wardrop—. [Here follows Mr. Wardrop's account at full length.]

*Sir J. Scarlett.*—It is not necessary to go through the whole; the details are of no importance.

*Lord Tenterden.*—Do you wish any more of it read, Mr. Brougham?

*Mr. Brougham.*—This is the one in December. I shall have occasion to read a sentence more out of it, which my friend will consider as read now.

*Sir J. Scarlett.*—I may have occasion to have another part read than you know. You had better do it now.

*Mr. Bellamy.*—What page?

*Mr. Brougham.*—491.

*Mr. Bellamy.*—That is before the commencement of the article; the article is No. 15.

*Mr. Brougham.*—Look at 491, article 2d, Medico-Chirurgical Transactions; then they examine the articles of that book, one after the other, but it is one review, as it were. If your lordship will look at it, you will see how it is. There is no doubt about it—it is all one article; it is headed Article II. in large Roman numerals. [The book was handed to his lordship.] Your lordship sees it is a review of a series of papers, one after the other, forming part of article the 2d of the Medico-Chirurgical Transactions.

*Lord Tenterden.*—I do not see it has any connexion with it.

*Mr. Brougham.*—It is the introductory part of the review.

*Lord Tenterden.*—You have a right to read any thing contained in the publication, by and by, as your evidence, it is quite clear.

*Mr. Brougham.*—I only submit to your lordship, it is the introductory observations to the article,—part of which article my friend is reading. It is quite clear it is so. It says, 'we have omitted certain papers in our last review.'

*Lord Tenterden.*—What has that to do with the article in question?

*Mr. Brougham.*—It is one part of the article; it is all part of Article II.

*Lord Tenterden.*—Then comes No. 13.

*Mr. Brougham.*—No. 13 does refer to Dr. Macleod's book; it is as if he quoted a page of the book he is reviewing. 13 and 15 are the numbers of the papers in the Medico-Chirurgical Transactions; that is the book under review at that moment; instead of quoting page such a thing of the book, he quotes article such a thing of the book. The article of Dr. Macleod is Medico-Chirurgical Transactions, of which this thing forms separate parts.

*Lord Tenterden.*—What do you say, Sir James Scarlett?

*Sir J. Scarlett.*—I apprehend my friend has no right to do it.

*Lord Tenterden.*—I cannot make it out to be part. I have read it.

*Mr. Brougham.*—My friend is reading part of a review.

*Lord Tenterden.*—That is true—suppose it was a newspaper. Any thing that is connected with the part that they select, you have a right to make them read as their evidence; but if it is not part of that, you may read it yourself by and by.

*Mr. Brougham.*—I shan't keep it up one moment longer, my lord. I have not made myself understood. Suppose a book consists of ten pages, and another book consists of a review of those ten pages: then the Reviewer says, the first page contains so and so; the second page contains so and so; then he comes and says, page five—page five of this book contains so and so;—then my friend reads what he pleases of page five, and he won't let me read the other.

*Lord Tenterden.*—Yes, he will, if you do it yourself. It seems to me to have as little connexion with article 15 as it has with any thing that appears in the next number of the Journal.

*Mr. Brougham.*—It is the introduction to article 15, as well as all the rest. It is the introduction of the whole of it. He says, we left out article 15 in the former number, and now we publish it.

*Lord Tenterden.*—It does not say a word about article 15.

*Mr. Brougham.*—"We are accused of treating certain papers as unimportant, because we have not taken any notice of them in our review of this book: we now mean to supply that defect, and for that purpose intend to take notice of those papers;" and then he takes notice of it.

*Lord Tenterden.*—I do not read it with any sense like it.

*Sir J. Scarlett.*—I am not bound to read any detached part: it will come from my friend, not from me.

*Lord Tenterden.*—I am satisfied it is not so connected with it.

*Mr. Brougham.*—Your lordship has my objection to that decision, which is that that is the introduction of this very thing that my friend is reading.

*Lord Tenterden.*—I think it is not: it is a general observation on the conduct of the work, having no more connexion with this particular article than it may have with any other article.

*Mr. Brougham.*—This is not the defendant's; this is Dr. Macleod's.

*Lord Tenterden.*—I know it.

*Mr. Brougham.*—It is a general remark of his own, on having left out certain papers.

*Lord Tenterden.*—That you may read as your evidence;—it does not connect itself with a particular article they desire to have read; it is general matter; it relates to any thing that may come at any time, for aught I know.

*Mr. Brougham.*—This is one of the articles.

*Lord Tenterden.*—I do not find any mention of aneurism in article 15.

*Mr. Brougham.*—He begins the next number 12. He says, we left out the following papers in our former account of that book.

*Lord Tenterden.*—I do not see that.

*Mr. Brougham.*—"In concluding our account—"

*Sir J. Scarlett.*—Don't read it.

*Mr. Brougham.*—My lord cannot understand my argument without my reading it.

*Sir J. Scarlett.*—Nobody is objecting to your giving the evidence: the only question is, whether I am bound to read it.

*Lord Tenterden.*—I think it is not: I am quite satisfied it is not so.

*Sir J. Scarlett.*—Now there is the next—April 1827.

[The account of the dissection of Mr. Wardrop's second case was then read from the London Medical and Physical Journal.]

George Gilder, *sworn, (examined by* Mr. F. Pollock.)

Q. Do you know the defendant Wakley?—A. I do not, sir.

Q. Have you an admission from his attorney that he is the editor of the Lancet? I will put this in first, my lord.

*Mr. Bellamy.*—Signed by Mr. Fairthorne, the defendant's attorney.

*Mr. Pollock.*—It may be taken as admitted.

*Mr. Bellamy.*—"I hereby admit the above-named defendant, that is Thomas Wakley, to be the editor of a certain periodical publication called the Lancet, mentioned in the declaration in this cause, and consent the above-named plaintiff shall not be required to give evidence thereof on the trial of this cause."

[Here the libel was read from the Lancet.]

*Sir J. Scarlett.*—The next number—the very last.

*Mr. Brougham.*—With great submission, as to the last number my objection is—I put it for your lordship's opinion as to the first objection I had to it, of its being since the matter. My other objection is, there is no proof whatever of the defendant on the record being the author of it. The date of the admission is the 13th of February, 1828. You must begin by proving us the author; you cannot take any thing that is called the Lancet, and read it.

*Sir J. Scarlett.*—My lord, I submit that it never could have been the intention of so *ingenuous a person as the author of the Lancet* to deceive any body by an admission; and, as he admitted he was the editor, and as this was bought at the same shop where the other was bought—"

*Mr. Brougham.*—You have not proved it.

*Sir J. Scarlett.*—The witness proves they were bought at the same shop. I apprehend it lays on him to show he ceased to be the editor before that day, How can such an admission mean—how can it be intended, to save expense and trouble to the parties, unless it is intended to cover all cases?

*Mr. Brougham.*—We meant to admit, and undoubtedly did admit, that we were the authors of whatever is set out in the declaration, of which alone we had notice. You may bring any thing on us after the declaration, according to your practice.

*Lord Tenterden.*—I think, Sir James, the admission don't comprise this—13th of February, 1828, which was before that Saturday;—it is only this: he is the editor of a certain periodical publication, called the Lancet, mentioned in the declaration, and consent the above-named parties shall not be required to give evidence thereof on the trial of this cause. I think that does not go to admit he is the editor of any publication of which the plaintiff has not complained in his declaration.

*Sir J. Scarlett.*—Will your lordship allow me? The publication is periodical; the word "periodical" is mentioned in the declaration: it implies a publication published at certain periods, and, as the declaration sets out only one, the admission certainly cannot mean to refer to that one only:—that would be, he was the editor of that number mentioned in the declaration. *It is a general admission he was the editor of that periodical publication.*

*Mr. Brougham.*—Of that day.

*Sir J. Scarlett.*—He meant the periodical publication generally—surely, my lord! *Shall an attorney be allowed, when he makes an admission, to use that admission to prevent the party from bringing evidence to prove a fact which is fairly within the meaning of the admission,* "I am the author and the editor of that periodical Journal?" Would not any attorney alive conceive that was sufficient to dispense with the necessity of his calling any witnesses to prove he was, at any time subsequent to the date of the declaration, the editor of that Journal? I apprehend it must be so, unless it was intended to deceive,—it must have had that meaning. I can carry it farther, and show that, up to last Saturday, the defendant's attorney admitted he was the author of that publication;—that he was the author.

*Lord Tenterden.*—Any further evidence you offer I will receive, before we decide it.

Mr. ROBERT GAMLEN, *sworn, (examined by* Sir JAMES SCARLETT.)

Q. Did you see Mr. Loftus, the defendant's attorney, last Saturday?—A. I did.

Q. Was that after the book was purchased—that last number?—A. It was.

Q. What passed between you and him?—A. I showed him the admission which has been put in, and asked him to admit it to be his admission in my presence, to prevent the necessity of its being proved—to prevent the necessity of the editorship being proved.

*Sir J. Scarlett.*—Your lordship has got that number, which was bought at the same shop where the other was bought.

Q. (*by Lord Tenterden.*)—You showed him his admission, and asked him what?—A. I showed him the admission, and asked if he would admit that to be his hand-writing at the end of it.

*Sir J. Scarlett.*—My lord, I can carry it a little further still.

Q. Is that the hand-writing of Messrs. Fairthorne? [Handing a letter to the witness.]—A. I cannot say.

Q. Is it a letter received by you from Messrs. Fairthorne, relating to this cause?—A. Yes, and replied to immediately.

*Mr. Bellamy.*—Addressed to Messrs. Poole and Company, and it is signed Fairthorne and Loftus, dated the 21st of June, 1827. "Wakley, at the suit of Macleod. In making the admission we have made—"

*Mr. Brougham.*—The date?

*Mr. Bellamy.*—The 21st of June, 1827.

*Sir J. Scarlett.*—It goes to the point.

*Mr. Bellamy.*—"In making the admission we have made, that the defendant is the editor of No. 194 of the Lancet, we felt nothing more was necessary, because we understood that to be the number of the Lancet which contained the article complained of by the plaintiff; but, as you require an admission that our client is *editor of the Lancet generally, we do not object to go that length, and now admit that he is,* although you will understand that it is usual, in cases of this description, first to see the plaintiff's declaration."

*Sir J. Scarlett.*—I appeal to your lordship, whether the attorney is not bound in this case, after that letter and that admission, to consider himself as having admitted it until he can show any change of the editorship.

*Mr. Brougham.*—My lord, with great submission—if your lordship calls on me to make an observation on this—up to the 21st of June I am bound by that admission—I am bound not only to admit him the editor of the 194th number, or whatever it was in the declaration,—although that is an admission fairly and candidly given before seeing the declaration, as it states; but also that he was generally the editor up to that time; but he may have ceased to be the editor, as Mr. Bacot ceased to be the editor, the day after, and consequently that does not carry the case a step further. The Lancet is conducted under an editor; it is sold at a particular shop; and, whoever the editor is, it will be continued to be sold at the same shop, just as well after the time he ceases to be editor as while he was editor. So that if the present defendant, Mr. Wakley, had ceased to be the editor the day after the last admission, or the day after the admission now in your lordship's hand, of the 23d of June, it would still continue to be sold at the shop, just as the Medical and Physical one published by Dr. Macleod, as is in evidence already, is now edited by him, and ceased to be edited by Mr. Bacot. I submit to your lordship, the proving a person to be an editor at one time is not to be taken as proof he is generally editor up to this time: you cannot. According to my friend's argument, five years hence I should still be concluded by this admission, because I admitted I am an editor up to a certain day; for, says he, it means this or nothing: that throws on you the disproof of every thing that is published in that paper at any future period, because, up to a certain day, you have admitted you were editor *non constat.* That this gentleman sitting before me, Mr. Fairthorne, the attorney for the plaintiff,—if he had been asked, "You have admitted, on the 13th, you are editor up to this time, will you admit you may be editor next Saturday, or will you allow us to give whatever is in the paper next Saturday in evidence?" In all probability he would say, "I cannot."

*Sir J. Scarlett.*—In the ordinary case of a newspaper, you show an affidavit made ten years ago—you produce a newspaper—

*Mr. Brougham.*—An Act of Parliament.

*Sir J. Scarlett.*—On whom does the *onus probandi* lay of showing the change—on the party?

*Mr. Brougham.—Until you swear you are not, you are to be held an editor.*

*Sir J. Scarlett.*—That is to show you have not sworn. Here is a general admission he is editor—published in the same form—bought in the same shop—he has made an admission.

*Lord Tenterden.*—I do not think I can consider the admission as extending to matters that have been published after the admission. I cannot do that. Is that your case?

*Sir J. Scarlett.*—Will you allow me for a minute—if your lordship will allow me to see if I can supply that which I think is very important.

*Lord Tenterden.*—By all means: I do not wish to hurry you.

*Sir J. Scarlett.*—If I can show—I cannot bring down by any evidence I am now possessed of,—although I clearly should have been possessed of it, but for that admission;—if I can show he has been the admitted editor down to a period much later than this action, whether that would make any difference in your lordship's judgment?

*Lord Tenterden.*—I think not. The letter goes later than the action brought—the admission of February goes down to that period. Going down as late as that, still I cannot consider it as going further.

*Sir J. Scarlett.*—I am very sorry for it.

Mr. Brougham then proceeded to address the Court and Jury, on the part of the defendant, as follows:

May it please your Lordship; gentlemen of the Jury; In that ornamental part of my friend's speech, in which he was pleased to make allusions to me, and which of course is now to be dismissed as mere ornament of that speech, he talked of a number of persons being assembled to hear an attack made, which he spoke of—an attack on Dr. Macleod. I am sure if there is any body who is interrupting his usual and useful avocations by any such expectation of what is to pass here in defence of Mr. Wakley, for whom I am Counsel, he had much better go hence and depart till required another time; because whatever my friend may intend to do here, or whatever pains he may have taken to collect persons to hear his panegyric on that respectable gentleman Dr. Macleod, I am sure I am not going to gratify any enemy of Dr. Macleod's, if he has an enemy—I know not that he has, except those who comment fairly upon his public conduct in the exercise of their duty of a similar kind with Dr. Macleod's, and as editors. I am sure nothing that shall fall from me will gratify any spiteful or unpleasant feeling, should it be harboured by any one individual, with respect to Dr. Macleod. I am about to call your attention, not to Dr. Macleod's conduct, but to the conduct of my client, for that is in issue before you, and only in issue before you: I am about to shew you, Mr. Wakley being the editor of a journal, which my friend justly observed has great circulation, the Lancet, has only acted, as any other editor would do, by what he also, I believe very justly, calls a rival editor; they do not always treat one another with that degree of meekness, and that degree of courtesy which my friend desiderates, when he says, "they are rival editors, and they are squabbling together;" but although they are rival editors, I quite agree with him, and therefore I took it down, "though they be rival editors, that is no reason why they should not treat each other with civility." No more it is: if there was a Court of Honour, Politeness, and Civility, in which my friend would be exceedingly fit to sit as Chief Justice, just as he would be to sit in any Court of Law—I know no man better qualified to exercise either function; he would call that man before him, and severely reprimand him for his incivility—if he were to say, "I am a rival editor;" "but," he would reply to him, "if you be a rival editor, that is no reason for your using him with incivility, and therefore I award you to make him three bows, and go out of the room!" But Courts of Law don't act in that way—Courts of Law don't punish and fine people for incivility, and don't give damages for incivility; for, if they did, the Juries would labour much more in their avocations than they now do; and then any man who was jostled in the street, or any man who did not take off his hat when another met him, or another who called him by a name without giving him, as it is termed, a handle to it, or any man who called another "Roderick," without saying "Doctor," (which really, I must say, does seriously appear to be the foundation of all this,—it lurks at the root of all this extreme disposition of vindication and revenge, and punishment of the defendant, and which glows through my friend's instructions, and therefore appears justly reflected in his speech from those instructions,—it is at the bottom of it,)—and every man who acted as I have described, would be called before the Court, and my friend would say, "I order you to call him, instead of "Roderick," instead of that, "Dr. Roderick," three times, in a loud voice, where the gold pill rears its head aloft in Warwick Lane, or whither they have transferred themselves, by migration, to the West-end of the Town; you shall in a proper time and place, between the hours when Physicians do chiefly haunt there, between the hours of twelve and one in the morning, before the first cock, (which they are known to sacrifice to their grave god,) between those hours, at the first cock, you shall in the name of Esculapius, your patron deity, call three times with a loud voice, "Dr. Macleod,"—what the French call an honourable amends for having called him "Roderick," without the word "Doctor!" So it would be, if such a Court of Honour there was; but Courts of Law have nothing to do with such things. Surely a man is not to get damages for saying "Roderick;" I should expect damages for a person calling me "friend," instead of my "learned friend," which is the courtesy of the law, and which is used towards us elsewhere by laymen. I do not deny these are rival editors: I agree with him they are rival editors; in the exercise of their rivalry, they have not treated each other with perfect civility—that is no reason why you are to be called on to award damages to the plaintiff, as if a serious wrong had been done to his private and personal character. I say nothing about this Lancet, (my friend has said a great deal about it, and a great deal about their own Journal,) except that I understand from Doctors it is a publication which has rendered great service to that useful and necessary profession. I happen to have very little acquaintance with Doctors; I never desire to extend it, unless in an unprofessional way; the less one sees of them the better, except as friends: but I have heard, from the little intercourse I have had occasion to have with them, that this is the character of the publication.

Now, gentlemen, as to its misdeeds, which my friend has talked about today, it really amounts to this: the charge is this,—Mr. Wardrop, an eminent surgeon, appears to have performed several cures, as he thought, and as others in the medical world appear also to have thought, but it was a subject of controversy; controversies, in most branches of art and science, are of great use to the public, for, by the conflict of opinions, light is elicited, by which the public benefits; and, in a matter of such infinite importance to the well-being of the community as the science of the healing art, the science of medicine, nothing can be more important and more essential to our interests, than that the cures that are from time to time adventured by bold practitioners should be discussed, and by being canvassed should be sifted as to their merits, lest it might be thought that the cure has been effected by a bold operation, which in fact never had been effected; that the operation had been followed by the death of the patient, for instance; or that the operation had been supposed to be on a disease of a particular kind, when in truth it was on a different disease,—in short, that the whole evidence on the case, on either side, may be brought forward, and the truth sifted on an ample and fair discussion. Then, so far from blaming the freedom of this controversy, I, for one, and I believe my learned friend would agree with me in the general, although he will differ, of course, owing to his instructions, which are not his opinion,—in the application of them to the present case, a controversy had arisen on this subject. It appears that Dr. Macleod, a respectable physician, and the editor, the rival editor, of a rival Journal, was inclined against, rather against the soundness of that view of the subject; and accordingly he publishes, in one of his Numbers, an account of the dissection of one of the cases of aneurism, in which the carotid artery was "supposed," was "supposed" to have been tied; clearly shewing he differed from Mr. Wardrop, for Mr. Wardrop's account, of course, would be a very different one; Mr. Wardrop's account was, that *he had* actually tied it. I will read you what my friend has actually read, to shew he differed from Mr. Wardrop,—"Case of carotid aneurism successfully treated by tying, (not by "supposing" you had tied,) by tying the artery above the aneurismal tumor;" that is what Mr. Wardrop says: the Doctor says, "The dissection of one of the cases of aneurism, in which the carotid artery,"—one of the cases.

*Sir James Scarlett.*—You are reading it from your own publication; ours says, "one of the recent cases."

*Mr. Brougham.*—What is the page?

*Lord Tenterden.*—"Dissection of one of the cases of aneurism, in which the carotid artery was supposed to have been tied,"—that is in it.

*Mr. Brougham.*—I am reading from the Number they put in; from the libel in question, supposed libel. What is your page, in April, tell me?

*Lord Tenterden.*—316: it is exactly transcribed.

*Mr. Brougham.*—So I thought, I thought I was right; just transcribed; I was quite sure I was right. Now "The dissection of one of the cases of aneurism, in which the carotid artery was supposed to have been tied." Now that, you see, implies that—in all the cases, the question in the profession is, are these cases of carotid artery, in which there is the aneurism of that important artery; has it been tied from the tumor, further off from the heart than the tumor, that is the question? He clearly shews he doubts that, which he had a right to do, by calling them "supposed" cases; that shews the meaning of Dr. Macleod: that Dr. Macleod takes one side of the controversy, and Mr. Wakley the other. Then comes the other, which also shews it, he says,—at the end of it, he says—"We are quite aware that mistakes will sometimes happen, even in the hands of skilful Surgeons; and it is this consideration which has induced us," now this comes in italics, "to withhold numerous other instances of unfortunate operations which have been transmitted to us." So that is in italics; although he does not tell them, he does not tell you, the cases, observe that! The question is, whether Mr. Wardrop is right; and whether his case and Mr. Lambert's case succeeded. He says they are "supposed" to have tied it beyond the tumor. He then gives a long discussion of that particular case, and then he comes and says, "we could apply that to a number of other cases which we have from private correspondents, but we won't,—a number of other cases of unfortunate operations."

*Sir James Scarlett.*—Not of that description.

*Mr. Brougham.*—Then what is the meaning of this? Because the question is of carotid artery; do you mean to say he means to refer to cases of trepanning, or of the stone? No such thing! This must be taken clearly in contrast with the subject matter of our article; the word "supposed" shews it, and the words "unfortunate operations," which are printed in italics. It does not mean he had cases about trepanning, it means he had numerous other cases of unfortunate operations of the same sort.

*Sir James Scarlett.*—He does not say of the same sort.

*Mr. Brougham.*—I will read the passage, and you shall see it.

*Sir James Scarlett.*—I read it distinctly.

*Mr. Brougham*—It was not read in going over the proof.

*Lord Tenterden.*—The words "of the same sort" are not here.

*Mr. Brougham.*—I am not saying so. "We are quite aware,"—this is what he says; "we are quite aware that mistakes will sometimes happen even in the hands of skilful surgeons, and it is this consideration which has induced us to withhold numerous other instances of unfortunate operations which have been transmitted to us for the purpose of publication, because they have not, like the present case, been connected with any important practical question:" so that he does not give those numerous other instances; but still observe what he calls them—"Other," other instances of unfortunate operations, clearly shews that he considers this as an instance of unfortunate operation. Now observe, and do not lose sight of this; the whole discussion which I am about to call your attention to in the Lancet, is an answer in detail to the various arguments which precede this: the answer is meant to rebut the presumption that this is a case of unfortunate operation, and the answer is meant to rebut the assumption that these cases of aneurism tied beyond the tumor, are "supposed" cases, because Mr. Wakley, or whoever is the author of this, which he is editor of, goes in detail through all those particulars, for the purpose of shewing that fact, and none of them prove this was a case in which the operation had failed, in which the party had died owing to the operation, or in which the operation at all could be treated, connected with the case in question, as unfortunate, and as shewing that there was any defect in Mr. Wardrop's method; that is the proof given in the whole argument. The one party, Dr. Macleod, relies on his comments—on those observations—on those operations which he gives. The other party, namely, Mr. Wakley, relies on his answer to this exposition of that proof—of the non-application to the point in dispute—of those commentaries of Dr. Macleod: now, that is the case between the parties. Now the charge brought against my client, the defendant, is this: that he accuses Dr. Macleod of having suppressed, willfully omitted for some months, the inserting of successful cases, and waited until an unsuccessful case came; or till a case which could be construed, or by reasoning perverted, into an instance of want of success, was in his possession; and then for the first time giving it: that is the question, as my friend says, and I do not deny that, in point of fact, this proceeded on a mistake: I

do not deny it. I do not at all deny that before the time when Dr. Macleod had published the account of the unsuccessful operation,—I do not deny at all, before that time, there had appeared an account in Dr. Macleod's publication of the other operation. I do not at all deny that: but my friend says that this must have been known to Mr. Wakley; and that he knowingly and wilfully published what he must have known to be an untruth, for the spiteful purpose of charging Dr. Macleod with a knowing and wilful omission. That is the charge against him. I shall shew you that is not grounded in truth. Is it to be conceived that any man would write in the plain undisguised terms which are used here in the following sentences: "Dr. Macleod is a disingenuous person, has duplicity as an editor, is an unfair controversialist, is an uncandid disputant in the medical arena, because he suppresses in his publication all mention of the cases before the other day, and then only gives the following case, and does not publish the successful cases." Is it to be believed that a man, be he ever so spiteful, unless he was deranged as well as spiteful; unless, instead of being the enraged physician, he was fitter for being the patient of a sane physician; unless he was absolutely a madman; would he have ever published to the world what he must know every human being who read the Lancet must have been aware was untrue, and which, therefore, could only injure himself, and not his supposed antagonist, the other rival editor, Dr. Macleod? If, I say, "A. B. went privately and did so and so," or "A. B. privately suppressed his knowledge of a fact;" a steward, for instance, suppressed his knowledge of certain gains coming to his master, or certain facts which his master ought to have known, that is a very slanderous, that is a very evil insinuation, if it turns out to be false; because I may deceive the whole world when I say it, and I may take my chance of the world not knowing the fact, being a private and not a public thing I am speaking of: but if, I say, "A. B. the man in question, who publishes a monthly magazine, or paper, or medical journal, and in that journal he never has mentioned such a thing; in that public journal, and in that public manner, he says he has suppressed the thing, "he has never from that time to this published a word of such a case:" good God! I must believe what I say to do so, because it is a public matter; it is of notoriety, and every man who reads that Journal, and reads this publication respecting the Journal, must be at once able to contradict me, and, instead of injuring Dr. Macleod, I am only offering an injury to myself, either for inaccuracy or falsehood, one way or the other. Observe what my friend admits, very fairly: says he, "the Medical Journal, and the Lancet, are addressed to the same medical public, and are read," says he, "by the same parties." No doubt! that is just what I say; that is my argument; they are read by the same parties: the man who reads the Medical Journal once a month, reads the Lancet once a week; and Mr. Wakley knew that just as well as my friend, and just as well as Dr. Macleod: and when Mr. Wakley publishes this, in which he charges Dr. Macleod with never having published faithfully, in good time, the account he afterwards published; that, publishing the failing case, he never published the successful case; he knew he was addressing people who did not read the Lancet without the Journal; but he was addressing, according to my friend's own admission, the very selfsame class of readers who read the Journal, and must at once have been able to satisfy themselves he was in the wrong, if wrong it was. It is inconsistent: no man will suppose Mr. Wakley did not thoroughly believe what he said: what can be more easy to account for his not knowing it? what can be more easy? Observe this book; the Medical Journal comes out in numbers; observe that: my lord has decided I cannot go to another part of this article as part of my friend's evidence; consequently I have not gone to another part, and I confine myself strictly to this: but my friend has given evidence which clearly shews that, in treating of papers which you see appear in the transactions of learned bodies, large volumes, each volume furnishing matter of much comment, that they continue the article from one number to another, for he has given that evidence; it was part of his averment in one of the counts of his declaration: the leading count, that he continued the article in question, the

April article, from one number to another. Is it not very likely the December article was continued from number to number? Is it not very possible one number might have been read, and one number might have escaped Mr. Wakley? Is it not exceedingly likely at the time he wrote this, and said the Doctor has never published an account of this at all, that the fact was, he had not seen the Number in which he had published the account? May not a man see one Number and not another? May he not, when he finds the first Number he has ever seen not mentioning it, he having been looking out for it, but a Number having escaped him, and missed him, in which the thing was, he sees, time after time, paper after paper, in that book, the review of those Transactions, but never any mention of this; that one, in which it is mentioned, escaped him; and then comes for the first time to his knowledge, for the first time he sees an account, and that is of the operation in which the woman died, and that described in a way which he thinks, right or wrong, is untrue. We have no business to decide between them, the doctors differ, one thinks one, and another another thing; he is angry for his having taken an opposite opinion, and an opposite side of the case, and as he thinks maltreated *his friend Mr. Wardrop,* who made that cure which he, as a medical practitioner, was so partial to. Being prejudiced, and prepossessed by these feelings, he says,—"This is very unfair; this is foul play; this is uncandid; it is duplicity:" whereas, says my friend, he ought to have been more slow to blame. I do not deny it. He ought to have been more sober minded: he ought to have been more cautious before he blamed: he ought to have gone and seen all the numbers, and looked at every page of all the numbers, before he made a charge against Dr. Macleod. I do not deny that—it is incidental to controversy; it is the nature of it; and men, when their minds are heated with religious, medical, or political, or any other controversy, it is very well known that they are apt to lose the Christian in the polemic: those who ought to have the most of that feeling, professionally are known to be, almost proverbially I may say, those who ought always to shew the greatest meekness and candour, have the least. If you will allow such a person to have that proverbial bitter hatred in his compositions, in his controversies, you will also allow a little for a more practical purpose—I won't say more practical; but men, as long as they are men, are apt to feel that more than higher objects, and are very, very likely to indulge in a little of the same haste, and a little of the same animosity: the question is, whether it has gone so far as to amount to a libel? My friend says, he has been held up to ridicule from the beginning to the end. He first says, "the yellow Journal, by that exceedingly sagacious, active, and intelligent editor, Roderick Macleod." This is all wrong; it is a libel, says my friend, to call a man not sagacious, not active, and not intelligent. Then he afterwards talks, in the course of the controversy, of yellow fungusses, which are ejected; these are medical allusions, which you and I do not well understand, they are taken from the *materia medica*; the ejectment is very different from the one we know of: they have their ejectings; they talk of ejecting; the body refuses or rejects a thing, and ejects; so, he says, the yellow funguses are ejected: my friend thinks that is also an exceedingly offensive and disrespectful mode of speaking of a respectable antagonist.

Now I hear my friend say he does not care a farthing about that; then what is it he does care a farthing about? I shall go over the different things; and, if he will say "I do not care a farthing" for each as I go over it, we shall probably reduce the bill to nothing in the end. "An editor, who places all his reliance for support on the corrupt patronage of hospital surgeons, and who appears to hate, mortally hate, not only general practitioners, but all those 'factious and disaffected persons.'" Who must have been the man who said these persons were factious and disaffected persons? It is quoted: it is quoted.* Then, somebody else, not Mr. Wakley certainly, but somebody else; whether Dr. Macleod or not is unknown to me; it is somebody with

* These words are quoted, in the Lancet, in such a manner as to imply that they are Dr. Macleod's. Dr. Macleod never used either these words or any having a similar meaning, as applied to general practitioners.—(EDITOR.)

whom he is conflicting, and about whom he is speaking; this is a sort of *argumentum ad hominem*; he throws into his teeth all those "factious and disaffected persons" who have espoused their cause, and it is well known that Mr. Wardrop is one of those persons. Now, my friend says nothing could be worse, because Dr. Macleod is a physician in practice; we are glad to find today, notwithstand what has been written, that he enjoys the same practice he did before, the same lectures as well attended, as the evidence is, as they were before; and he is still the editor of this Journal, notwithstanding the attack on him in his editorial capacity, distinctly so and nothing else: but, says my friend, it is very hard for a practitioner, who depends so much on being in favor with general practitioners, to be held up as hating general practitioners, as, if he had said so of him as Dr. Macleod, if he had said so of him as a private individual, if he had said so of him as a physician, that is a very different case; but it is most carefully guarded from all such imputation—it is most carefully screened and protected, as I shall shew you by the very words made use of, from any possibility of the drawing an inference, or putting a construction on it like this; for he says, it is an editor, not a physician, but it is an editor who places his reliance on such support. Have I not a right, if I am editor of one journal, to say "my rival editor?" and my friend admits it is to be treated as rival editors conflicting on a controversy, "my rival editor looks to such a party." Have I not a right to say "my rival editor looks to such another party, in his capacity of an editor?" It is only in that capacity; he does not say, as a physician, "he hates the general practitioner;" he does not say, as a physician, he hates the surgeons; he does not say, as a physician, he rebels against the college; but he says "an editor who places all his reliance of support,"—on what? Not among patients! Not support, as a physician, in his profession of physician,—"for support in the patronage of so and so." Does not that mean the support to him as editor, by the taking in his Journal? I ask any man of plain common sense and understanding, whether those words are capable of any construction except that? When I say, "a physician places his reliance for support on such practitioners," that is one thing; when I say, "as an editor, he places his reliance for support on those practitioners, it is clear, decidedly clear, that I mean to say, the support of his Journal, as an editor; and I defy any other man, any one who applies his wit, for an instant, to construe the passage otherwise. It is utterly impossible that it can have another meaning.

There are many other observations respecting his editorial conduct; then he says, "for instance, to publish no account of the success." I have explained that already, because he had not seen it before.—"To publish no account of their success, and to fabricate cases of failure as soon as possible." What is meant by fabricate? As I have already shewn, I say that the whole article that precedes these words is a proof; and shews that this gentleman, Mr. Wakley, or whoever it is that writes it, is setting himself to demonstrate that a false conclusion had been drawn, in the page before, from the facts of the unsuccessful case. You will see one after the other, when it is read; as you must perceive if you take it with you and read it; you will find argument after argument, and topic after topic, replied to; and argument after argument urged,—to shew a false conclusion had been drawn from the facts of that case, for that it had nothing to do with the tying of a ligature to an aneurism, and, therefore, a false construction had been put on it by Dr. Macleod; it was false, in no invidious sense of the word: but he had fabricated a case, he had made it unsuccessful when it was not; fabricated in that sense of the word; and made it appear a case against Mr. Wardrop, when it was not such a case, or rather was one for him. That is the controversy,—God forbid I should call on you to say which was right! Mr. Wardrop may be wrong. Dr. Macleod may be perfectly right. What he means to say, regard being had to the whole of his discourse, is, that Dr. Macleod suppressed the case for some time that was successful: I have shewn you how that was, and that he mis-stated and mis-understood, mis-apprehended and mis-stated, the inferences from the case that was 'supposed' to be unsuccessful."

You are to say whether, taking all the matter here into consideration together, and reminding yourselves also that it is a conflict of those rival editors upon a matter in controversy in the medical world, which they pretend on either side to enlighten; one charges another in a certain way in the conduct of his journal, and in his editorial capacity merely, (for even when he talks of duplicity, it is editorial duplicity, and nothing else;) that he, as the one editor, charges the other editor with certain conduct, simply in the functions of an editor—that is all. Now, he being an editor, comes, like all authors, under the inspection and review of his fellows, rival editors, journalists, reviewers, call them what you will, who from time to time take part in literary politics, or professional controversies, and are accustomed, under the name of discussing and criticising each others' productions, to afford information to the public, or helps to guide the public taste. That the greatest latitude ought to be allowed to persons who so act I not only state as an obvious proposition, dictated by common sense and common reason, and the advantages of which latitude are the result of all and every day's experience, but I have the venerable authority of a most able and learned Chief Justice, who formerly sat in this Court, and who, certainly, I must say, with great humility, and the utmost respect for his memory, cannot, on any account, be accused of too great a leaning towards libellers and towards persons who had exceeded the due bounds within which discussion ought to be confined, and by which the feelings and passions of controversialists ought to be restrained; but my Lord Ellenborough, to whom I refer, was rather one who, from a natural indignation at slander, and every thing that could be termed libel and slander, felt, and from a great regard for the value of character, himself a man of the highest and most unsullied character, and feeling its value, he therefore leant, rather leant against than for those (I think I am putting it very fairly when I say so,) who exceeded those due bounds, and restrained themselves too little in controversy. And yet that learned Judge had the following case brought before him, and directed the Jury in such a way that the Jury found a verdict for the defendant, on the simple ground stated by his lordship, that it was not exceeding the bounds of due criticism; and he referred it to them to say, with a plain indication of his opinion, the bounds of due criticism had not been exceeded. A gentleman had published a book, just as Dr. Macleod edits a book, and publishes in that book which he edits; so had a gentleman, called a Sir John Somebody, it is needless to name his name again, had published a book respecting Ireland; and another person, not in a review, not called for as a reviewer, or by the constant duties of a journalist, as the gentleman who edits the Lancet may be, to give the medical world an account of whatever passes in it; but really, having nothing else to do but to laugh at his fellow authors, this person publishes a book called "My Pocket Book, or Hints for a Ryghte Merrie and Conceited Tour," in quarto, to be called "the Stranger in Ireland." There was a distinct allusion to a book called "The Stranger in Ireland," which that knight had published, and he brought his action; he called two noble persons, who proved they would have bought the book, and many others might have been taken to be in the same situation, if it had not been for this exceedingly ludicrous composition, which was the attack which my friend would call a scarifying, a bitter, ridiculous, and sarcastic attack on that gentleman's production, so that the sale of that book had been injured. So far from the sale of Dr. Macleod's being injured, he is now sole editor, whereas, formerly, he was only joint editor; his employers are more and more satisfied with him, because the book is succeeding better than ever, that you may infer, (the publication was after he had been sole editor,) they have made no change in their arrangement since.

Now you will find, my friend says, "he only should talk of the book, and welcome," says he; but if he talks of the person, there is an end of the case, there is a libel, and you must find a verdict for the plaintiff. What is the case that is reported by a very learned friend of mine, with his usual accuracy, in the first volume of his Nisi Prius Reports—"By a knight errant, (thereby alluding to the said Sir John) and which same libel contained therein certain false, scandalous, malicious, and defamatory things, and, among other things, a print." Now, observe, we have never put a print, we have never turned an editor into ridicule by printing him; printing him we have, but not painting him. What do you think was the prin of Sir John Carr in this book? I wish no advantage to be taken of that, he is a worthy man, his book is an excellent entertaining book, and very useful to persons who go to that part of the United Kingdom, and he was made a knight by his Grace the Lord Lieutenant, very much on account of his literary merit—that I happen to know. This was the malignant and spiteful way in which that author attacked him; he writes a whole book upon him, not an article in a journal; he writes a book with a frontispiece, entitled "The Knight," meaning Sir John, "a Knight leaving Ireland with regret," that is the style of the frontispiece, "and containing and representing in the said print, a false, scandalous, and malicious, defamatory, and ridiculous, representation of the said Sir John, in the form of a man of ludicrous and ridiculous appearance, following the said representation of the said Sir John, and representing a man." This is the caricature, and it is so graphically described by the draughtsman who drew the declaration, I am sure his brother painter who drew the picture could not have brought the image better before you. "Loaded with, and bending under the weight of three large books, one of them having the word 'Baltic' printed on the back thereof"—that was a book, on the Baltic, Sir John had published; "and a pocket-handkerchief appearing to be held in one of the hands of the said representation of a man, and the corners thereof appearing to be held or tied together, as if containing something therein, with the printed word 'wardrobe' depending therefrom; thereby falsely, scandalously, and maliciously meaning and intending to represent, for the purpose of rendering the said Sir John ridiculous, and exposing him to laughter, ridicule, and contempt; that one copy of the said first-mentioned book of the said Sir John, and two copies of the said book of the said Sir John, secondly, above mentioned, were so heavy as to cause a man to bend under the weight thereof, and that his, the said Sir John's wardrobe was very small, and capable of being contained in a pocket-handkerchief."—Now you see that is the meaning. Can any thing be more exquisite than the pencil of the special pleader who has so well, not only given the picture of the author, but the meaning of the author; he has told you all that passed within the divine artist's mind—that great painter's mind who made Sir John represented so. Is that a fair review of a man's book?—is that a fair criticism of his book? You may say that is an attack on his poverty: neither personal deformity or poverty, God knows! is any shame to any man. He brings his action, objections are taken, and Lord Ellenborough says, "here the supposed libel has only affected that work of which Sir John Carr is the alleged author." Have we done more? I have shewn it is all as editor. If an author may be attacked, so may an editor; he is in the nature of an author. "And one writer, in exposing the follies and errors of another, may make use of ridicule, however poignant. Ridicule is often the fittest weapon that can be employed for such a purpose. If the reputation or pecuniary interests of the person ridiculed suffer, it is *damnum absque injuria.* Where is the liberty of the press, if an action can be maintained on such principles? Perhaps the plaintiff's Tour through Scotland is now unsaleable, but is he to be indemnified by receiving a compensation in damages from the person who must have opened the eyes of the public to the bad taste and inanity of his composition? Who would have bought the works of Sir Robert Filmer, after he had been refuted by Mr. Locke? But shall it be said that he might have sustained an action for defamation against that great philosopher, who was labouring to enlighten and ameliorate mankind? We really must not cramp observations upon authors and their works. They should be liable to criticism, to exposure, and even to ridicule, if their compositions be ridiculous, otherwise the first who writes a book on any subject," and so forth. Then he goes on further, all in the same tone. "And even the caricature does not affect the plaintiff, except as the author of the book which is ridiculed. The works of this gentleman may, for aught I know, be very valuable; but, whatever their merits, others have a right to pass their judgment upon them, to censure them, if they be censurable, and to turn them into ridicule if they be ridiculous. The critic does a great service to the public who writes down any vapid or useless publication, such as ought never to have appeared: he checks the dissemination of bad taste, and prevents people from wasting both their time and money upon trash. I speak of fair and candid criticism, and this every one has a right to publish, although the author may suffer a loss from it.—Such a loss the law does not consider an injury, because it is a loss which the party ought to suffer; it is, in short, the loss of fame and profits to which he was never entitled: nothing can be conceived more threatening to the liberty of the press than the species of action before the Court. We ought to resist an attempt against free and liberal criticism at the threshold."

Now, gentlemen, I humbly submit the case under his lordship's direction to you; it is for you; his lordship will leave it to you; it is as an editor, and in his capacity as an editor,—over and over again you are reminded of it; in his editorial capacity it is that the rival editor pronounces his opinion, and, proceeding on a mistake with respect to a fact, which must needs have been committed by him, because, as I told you before, no man breathing could have stated that which he knew every man breathing, or at least every man reading and seeing, could contradict him in; it could not by possibility be otherwise than a mistake: it is for you to say whether this is written of him in his capacity of author or editor, or both.

There was a case very lately in the Court of Exchequer, in which I was of Counsel for one of the parties, and which I afterwards moved in Court, and had the opinion of the Court upon, strongly confirming—I should say carrying it, if any thing, a step further, rather than falling a step short of the law as laid down by my Lord Ellenborough. An author had written a book, in which he says, "Mr. Such-a-one," who brought the action, the case of Hall and Longman, "Such-a-one gave a lecture the other day," he was a lecturer like the doctor,—"Mr. Such-a-one gave a lecture, and professed to have discovered a method, a certain method; he had great fluency, and he had great effrontery; he had some address, and some cunning; nothing seemed to be wanting to him but honesty, common honesty." I think it was "honesty," in so many words; "he takes my invention, which I had published long before, he tricks it out, and decks it out as his own, and he pretends to be the author of this, most dishonestly." Now the charge is of dishonesty, and of dishonesty of the grossest kind; namely, of having got up at the West end of the town, before two or three hundred people, and pretending to give a lecture and tell of a discovery of a method which the individual, a doctor, who was the author, and then the defendant, in that case, had published the year before; he takes it to himself, he is a man of impudence, he is a man of effrontery, he has every thing but honesty; but honesty, that quality which makes one man's property secure from another man, that quality this individual has not." The Jury found, under the direction of the learned Judge, there was no proof of special damage any more than here, and the Jury found, under the direction of the learned Judge, a verdict which satisfied him. I came to move the Court afterwards, they found for the defendant it was a criticism, or an attack on him in his capacity of an author, and no more. That must have been the ground. I moved the Court, and first I stated, even if it were an attack on him in his character of an author, it was charging him with what no man, I cannot separate the author from the man so completely as to know that a man can be honest as an author, and yet knavish as a man; I argued that the injury is imputed here of interfering with another man's property, whether done by an author publishing a book as his own, which is mine, or whether it is done by taking my property from me which he has no right to intermeddle with; still it is a charge of dishonesty in the one case as well as the other. The Court of Exchequer unanimously gave judgment *seriatim* against me on that point, and they maintained it was only a remark by one author against another, and that it was childish to think that Courts of Law were to be loaded with quarrels formed on such grounds, and refused to grant the rule.

Gentlemen, I put it to you, that the case which I have cited is stronger than the present. It is worse to say a man has effrontery and wants nothing but common honesty; and not to say that vaguely and generally,—although that would be bad enough; but to make that out by a particular case given, which case was admitted to be false, for they never pleaded a justification;—it was worse, a thousand times, to say that, than talk of editorial duplicity—to call a man "Roderick" instead of Doctor—to talk of yellow funguses, instead of books—to talk of the first of April in connexion with his name, instead of any other day of the year—to talk of fabricated in the way I have described to you he means fabricated, and can only mean it,—and to talk of wilful omission or omissions as if they had been wilful, when they were either wholly accidental or no omissions at all—that being a mistake.

Gentlemen, according to this case, this must be allowed too. I submit these observations to you, perfectly confident you will give them the attention which their intrinsic weight, and their bearing on the case, will appear to you to deserve; and so doing, I take my leave of you, hoping that nothing that I have said,—I am most anxious, after the ornamental little piece of fancy which my learned friend conjured up for your amusement today,—I am most anxious to add this much, trusting and hoping that nothing which I have said, from the beginning of my address to the end, will be taken or deemed by you, or any one who hears me, to be a reflection, in the slightest degree, against the respectable character of the plaintiff.

Lord Tenterden, in summing up:—

Gentlemen of the Jury,—This is an action brought by Dr. Macleod for a libel; the publication of which the plaintiff complains appeared in a medical periodical work called the Lancet, of which it is admitted the defendant is the editor, and for the contents of which he is therefore responsible. The matter of the publication relates to the plaintiff's conduct as editor of another medical publication, called the London Medical Journal.

Now, a man has an equal right by law to have his character as an editor protected, as he has a right to have his character as a professional man, or a man of business, or of any other kind. In remarking, however, and criticising on the works of literary men, whatever is fair and reasonable to be said respecting them, or their conduct, as connected with their works, may lawfully be done. But if any person goes beyond the reasonable line of fair and honest criticism, and makes an attack on the character of another in his editorial capacity, as in any other capacity, he is answerable in damages to the plaintiff who may be attacked.

The subject of the plaintiff's publication, in this instance, and also of the defendant's, appears to be the treatment of a disease called aneurism in the carotid artery. The learned Counsel, who has addressed you last, said a great deal about the existence of some controversy. I cannot find, in the publication, that any controversy at all did exist on the subject. It appears attempts had been made—some recently, and some at a more remote period,—to find a cure for that malady by tying up the artery beyond the place at which the tumor existed, it being often difficult to get at it on the side next the heart; and then the plaintiff, in this first number of his publication, speaks of that as a matter which, if it be practicable, is a great advance of science in the practice of surgery, if it can be done with effect, and that it would be much recommended if it could be done with success; but we know that, whether an operation can be performed with success in a particular manner, will depend on the result of many cases: there must be several instances and experiments tried, before you can come to a conclusion one way or the other.

Now, the plaintiff had, in a publication put forth by him as early as the month of December, 1825, given an account of a case of a cure of this disorder, called carotid aneurism, by Mr. Wardrop: *it is mentioned in the body of that*

***work, and it is mentioned on the cover also:*** " Case of Carotid Aneurism, successfully treated by Mr. Wardrop," referring to the page at which it is found, and in that page there is an account given of the treatment of a lady considerably advanced in life, represented and appearing to have come from Mr. Wardrop himself, and represented as having been successful ; and the editor, the plaintiff, says on that—" The result of the case has fully proved the legitimacy of this reasoning, and, as it is the first successful operation of the kind which has been placed on record, we should consider it a great omission were it not to be found in the pages of our Journal." Now, it is important for you, in the consideration of this case, to bear in mind that, as early as December 1825, the plaintiff's work contains an account of the treatment of a case of that kind by Mr. Wardrop ;—I say it is very important for you to attend to that, because you will find, when the defendant's publication comes to be more particularly mentioned to you, that he asserts, until the month of April 1827, the plaintiff had noticed no case of this kind : his assertion, therefore, in that respect ***is manifestly false.*** It is said that he must be supposed not to have known it was false—to have written under a mistake.

Gentlemen, before a man takes on himself to assert a fact respecting another, and to assert it in a way that may be prejudicial to him, he is bound to see that the fact that he asserts is true ; it is not for him to come afterwards, and say, " I made this assertion—I find out now that I was mistaken in it, and am sorry for it." It is hardly to be allowed; because in the mean time, you know, the injury is done to a man's reputation by the readers of the work. The plaintiff having published that in the month of December, 1825, he does not appear, in this publication, to have noticed any other case of that kind until you come to the month of April 1827, and then the publication contains this :—" Dissection of one of the cases of Aneurism, in which the Carotid Artery was supposed to have been tied beyond the Tumor."

[Here follows the account from the London Medical and Physical Journal.]

He says, the reason why I give you the account of a case in which this operation was supposed to have been performed, which turns out not to have been performed, is because it relates to an important practical question. Then comes the publication in the defendant's work, of which the plaintiff complains. It begins in this manner—" We must again return to the April number, for the purpose of noticing two papers which refer to the recent operations for the cure of carotid aneurism, by tying the vessel beyond the tumor. The first case successfully treated after this method was published by Mr. Wardrop—" (that is the case the plaintiff published in his Journal of December 1825,)—" in Part 1st, Volume 13, of the Medico-Chirurgical Transactions, on the 5th of January, 1825, and was copied into the Lancet of December the 31st, 1825 ;—the second case, treated by Mr. Wardrop, appeared originally in the Lancet of December the 23d, 1826 ;—the third case, treated by Mr. Lambert, was published in this Journal of March the 24th, 1827 ; during the whole of which period not one word has been said of these most important operations in the yellow Journal."—That is untrue ; because one of the first of them had been mentioned, and mentioned in a way complimentary to Mr. Wardrop, who had performed the operation,—" by that exceedingly sagacious, active, and intelligent editor, Roderick Macleod. But, on the 23d of March, the subject of Mr. Wardrop's second operation having died from an enormous hypertrophy of the heart—" Whether it was the subject of the second operation of Mr. Wardrop, or any other, does not appear by this publication of the plaintiff, but probably, however, it was.—

"Roderick, in his April number, presents his readers with his first notice of either of these operations—" Now, that is not so—"in the following account of what he calls the 'dissection' of the body. This account we will here insert, italics, capitals, and all, in fact, without altering either word or letter."—Then he furnishes the account of the dissection of the body, and declaring the artery had not been tied up as had been supposed.

[Here follows the libel.]

Now that is the matter of which the plaintiff complains. That there is a great deal of attempt in it to cast ridicule on the plaintiff is not denied. I think it can hardly be said, by any person, that there is not in it a great deal of bitterness; but if all this has been called for by the conduct of the plaintiff as the editor of the Medical Journal, I should say the defendant was entitled to your verdict; but, then, has it been called for? Now, you see almost the whole of what the defendant says here to disparage the plaintiff as an editor is this, to bring it to a short point, Cases existed, and were known, of successful treatment of this disorder; Dr. Macleod never once publishes any one of them; withholds all from his readers; as soon as a case occurs in which it is apparent there has been a mistake, he gives that to the world. *Was that true?* Dr. Macleod had, as early as April 1825, given an account of the very first operation in terms of compliment to Mr. Wardrop. When you find that the terms which are contained in this libel are bottomed on a *false assumption —for so they clearly are*,—it is for you to say whether they are not, in your judgment, calculated to injure the plaintiff's character as a medical practitioner, or in any other way, or whether they are fairly called for by any thing as regards his character: if you think they are fairly called for by what he had done as editor, find your verdict for the defendant;—if you think they are not called for, (and, as they are founded on a *false assertion*, I can hardly suppose you will think they are called for,) then the plaintiff is entitled to your verdict; and the amount of damages is for your consideration, only take care that they are of a moderate nature.

Verdict for the Plaintiff—Damages £5. *Certified.*

To the readers of this Journal, who must be acquainted with the line of conduct adopted by the Editor towards the *Lancet*, but few observations on the trial above detailed can be necessary. For several years, no notice of the *Medical and Physical Journal* was taken in the *Lancet:* suddenly, however, it was assailed by constant misrepresentation and virulent abuse. Extraordinary as this change may appear, it is easily accounted for. Anxious to render the publication more worthy of the patronage which it had received, we formed arrangements, in the spring of 1826, by which Cases and Papers were transmitted by some of the most eminent members of our profession in London, for insertion in the *Medical and Physical Journal.* The improvement of another periodical was obviously either hurtful to the feelings of the Editor of the *Lancet*, or perhaps he feared it might prove injurious to his interests; and from that hour we, and all who wrote in our Journal, became marked as the objects of obloquy and slander. Disdaining to retaliate on so illiberal an opponent, we remained silent, and bore with "a patient shrug" the succession of revilings which were heaped upon us in that peculiar strain for which the *Lancet* is so remarkable, but which would never have induced us to deviate from the path we had marked out. It was not to protect us from the vulgar abuse of the *Lancet*, that we appealed to a Court of Law; but it was to clear our character from an accusation, which, if true, would have rendered us utterly unworthy of the confidence of our readers, and unfit to continue the direction of a Journal, the good faith of which was never before called in question, and we are bold in saying has never in our hands been compromised.

The evidence afforded by the details of the trial will be sufficient to convince any one who takes the trouble to peruse it, that the accusations of the *Lancet* are wholly without foundation. It will be seen that we had published the case of Carotid Aneurism (which we were said to have suppressed) on the 1st of December, 1825, and bestowed upon the operation the humble tribute of our commendation. It is remarkable, too, that the *Lancet* did not publish the case till December 31st, nearly five weeks after it had appeared in the *Medical and Physical Journal.* The circumstance assigned by the Editor of the *Lancet* as having led him to suppose that the case in question had not been published by us, is this:—Mr. Wardrop's paper was contained in the *Medico-Chirurgical Transactions,* the review of which having extended through two Numbers, we stated that, instead of continuing to analyse what remained, we should *probably* insert the papers in our COLLECTANEA: but that department in the following Number being pre-occupied by the posthumous papers of the late Dr. Baillie, we resumed our analysis; a circumstance pointed out by two notices on the cover,—one referring to the *Medico-Chirurgical Transactions,* and the other to this particular case, which was likewise entered in the INDEX to the volume. To say that, because this case could not be found where we had said it would *probably* be placed, afforded any proof that it had therefore not been published at all, and thus misled the Editor of the *Lancet,* is a mere subterfuge, to which no credit can reasonably be attached.

With regard to the circumstance of omitting Mr. Wardrop's second case, and that of Mr. Lambert, our reason for having done so is this: we never have regarded the *Lancet* as a publication of sufficient authenticity to induce us to take any cases from it. Had we partially selected from that work, it might have been said that Mr. Wardrop's second case deserved a place as well as his first; but we have always, and for the reason above mentioned, avoided quoting from the *Lancet.* As to the account of the dissection of Mr. Wardrop's second patient, we assigned our reasons for inserting it at the time, namely, because, in a previous part of the Number, Mr. Travers had alluded to this mode of operating as likely to prove of advantage "if borne out by similar results," and because we regarded the case as one which involved a great question in the practice of surgery.

A simple statement of these facts in this Journal was not calculated to give them the publicity they have obtained from a Court of Law, and it was this consideration which induced us to have recourse to legal measures. We have to apologise for having devoted so large a portion of this Number to matters personal to ourselves, and therefore foreign to its legitimate purposes:—hereafter, it is scarcely necessary to say that we shall adopt the same course we have hitherto pursued; leaving to his nicknames and his ribaldry one who can never form a fair and honourable opponent in any scientific or literary controversy.

*** There is one circumstance connected with the trial, which does not appear in the above account of it, and to which we think it right to direct attention. In such cases, the plaintiff has it in his power to bring an action against the printer or publisher of the work, as well as against the editor, so as to double the costs; and this method, it may perhaps be remembered, was adopted by Mr. Wakley towards Dr. James Johnson. We directed our attorney to inform the defendant, that, if he acknowledged himself to be the editor of the *Lancet*, "for all the purposes of this cause," "so that the plaintiff may have no occasion to prove that fact on this trial," we would content ourselves with the single action. These terms were accepted; and Sir James Scarlett, guided by the honourable understanding which exists in such cases, regarded any proof of the editorship as unnecessary, seeing the admission was a general one, made in acceptance of the above-mentioned conditions. The admission was dated February 13th, and the editorship of the *Lancet* published three days after was denied. The objection was held good in law, and made a material difference in the features of the case; but we do not envy those who made it the indignant remarks of Sir James Scarlett on the occasion. This we trust will prove a caution to any other who may be placed in similar circumstances to ourselves.

### METEOROLOGICAL JOURNAL,

By Messrs. Harris and Co. Mathematical Instrument Makers, 50, High Holborn.

| January | Rain gauge. | Moon. | Thermom. | | | Barometer. | | De Luc's Hygrom. | | Winds. | | Atmospheric Variations. | | |
|---|---|---|---|---|---|---|---|---|---|---|---|---|---|---|
| | | | 9 A.M. | MAX. | MIN. | 9 A.M. | 10 P.M. | 9 A.M. | 10 P.M. | 9 A.M. | 10 P.M. | 9 *a.m.* | 2 *p.m.* | 10 *p.m.* |
| 20 | | | 51 | 53 | 43 | 30.13 | 30.18 | 87 | 90 | W | SW | Fair | Fine | Fair |
| 21 | | | 47 | 50 | 45 | 30.13 | 30.11 | 94 | 96 | WSW | SW | — | — | — |
| 22 | | | 45 | 46 | 40 | 30.00 | 30.13 | 96 | 96 | WSW | WNW | — | — | — |
| 23 | | ☽ | 48 | 52 | 44 | 30.18 | 30.24 | 97 | 98 | W | NW | — | — | — |
| 24 | | | 50 | 52 | 48 | 30.23 | 30.23 | 97 | 98 | WSW | WSW | Cloudy | — | — |
| 25 | | | 53 | 54 | 42 | 30.20 | 30.01 | 98 | 98 | WSW | WSW | — | Rain | — |
| 26 | | | 45 | 51 | 47 | 30.15 | 30.23 | 94 | 96 | WNW | W | — | Fair | — |
| 27 | | | 49 | 53 | 37 | 30.24 | 30.31 | 94 | 96 | WNW | W | — | — | — |
| 28 | | | 42 | 47 | 42 | 30.31 | 30.25 | 90 | 96 | W | WSW | — | — | Cloudy |
| 29 | | | 45 | 50 | 40 | 30.08 | 30.06 | 96 | 98 | SW | W | — | — | Fair |
| 30 | | | 43 | 47 | 40 | 29.98 | 29.90 | 96 | 98 | W | SW | — | Fine | — |
| 31 | | | 45 | 49 | 43 | 29.90 | 29.86 | 98 | 98 | WSW | SW | — | — | Rain |
| Feb. | | | | | | | | | | | | | | |
| 1 | .3 | ○ | 50 | 54 | 47 | 29.95 | 29.96 | 98 | 98 | W | W | Rain | — | Fine |
| 2 | | | 50 | 51 | 39 | 30.01 | 30.08 | 96 | 90 | WNW | NW | Fair | — | — |
| 3 | | | 45 | 49 | 39 | 30.34 | 30.29 | 96 | 97 | WNW | W | — | — | — |
| 4 | | | 45 | 52 | 39 | 30.24 | 30.13 | 94 | 98 | NW | W | — | — | Cloudy |
| 5 | | | 46 | 53 | 48 | 30.15 | 30.01 | 93 | 98 | W | NW | Cloudy | Fair | — |
| 6 | .10 | | 50 | 54 | 45 | 29.93 | 29.91 | 98 | 98 | W | NW | Rain | — | — |
| 7 | .12 | | 51 | 54 | 41 | 29.84 | 29.77 | 94 | 99 | NW | W | Fair | — | Rain |
| 8 | | ☽ | 44 | 47 | 39 | 29.76 | 29.69 | 90 | 99 | NE | NE | — | — | Cloudy |
| 9 | | | 43 | 46 | 34 | 29.63 | 29.63 | 90 | 98 | NE | NE | Cloudy | Rain | — |
| 10 | | | 35 | 36 | 30 | 29.71 | 29.54 | 95 | 98 | ENE | SE | Cloudy | Cloudy | — |
| 11 | | | 32 | 33 | 28 | 29.35 | 29.73 | 95 | 90 | ESE | NE | Snow | Snow | Fair |
| 12 | | | 29 | 33 | 28 | 29.83 | 29.93 | 93 | 94 | N | N | — | Fair | — |
| 13 | | | 30 | 32 | 32 | 30.01 | 29.92 | 93 | 98 | NE | SE | Fair | — | — |
| 14 | | | 33 | 35 | 32 | 29.45 | 29.43 | 96 | 98 | S | W | Snow | — | Cloudy |
| 15 | | ● | 34 | 38 | 31 | 29.62 | 29.72 | 98 | 98 | NW | WNW | Fair | — | — |
| 16 | | | 32 | 36 | 31 | 29.76 | 29.74 | 94 | 92 | WNW | SW | — | — | — |
| 17 | | | 34 | 38 | 32 | 29.61 | 29.44 | 90 | 94 | SW | SSE | Fine | Fine | Fine |
| 18 | .14 | | 36 | 41 | 36 | 29.29 | 29.21 | 97 | 98 | SE | W | Cloudy | Rain | Rain |
| 19 | | | 38 | 47 | 37 | 29 26 | 29.26 | 99 | 98 | WNW | WSW | Cloudy | Fine | Fine |

